# A culture-informed psychological model for elite Chinese mountaineers: an exploratory study and development of a performance-based assessment tool

**DOI:** 10.3389/fpsyg.2026.1746906

**Published:** 2026-03-25

**Authors:** Kexiang Yang, Ge Chen, Rong Fan

**Affiliations:** 1 Department of Physical Education, Jeonbuk National University, Jeonju-si, Jeollabuk-do, Republic of Korea; 2 College of Physical Education, China University of Geosciences (Wuhan), Wuhan, China; 3 School of Basic Medical Sciences, Shandong University, Jinan, China

**Keywords:** Chinese mountaineers, elite athletes, four-factor model, high-altitude mountaineering, performance enhancement, psychological assessment, willpower

## Abstract

**Introduction:**

The psychological determinants of performance and survival in elite high-altitude mountaineering remain poorly understood, partly due to the difficulty of recruiting substantial samples from this highly specialized population and the limited applicability of Western-derived frameworks to culturally distinct cohorts such as Chinese mountaineers. This study aimed to develop and preliminarily examine a culture-informed psychological model for elite Chinese mountaineers and to establish a performance-based evaluation framework.

**Materials:**

Using a cross-sectional exploratory design, 15 psychological indicators were assessed in 84 Chinese mountaineers (53 elite; 31 non-elite) using validated Chinese-language instruments. Independent-samples t-tests were conducted to identify performance-discriminant indicators, and principal component analysis (PCA) was employed to explore the underlying psychological component structure.

**Results:**

Elite mountaineers scored significantly higher on 10 indicators, including Perseverance (*d* = 0.66), Goal Clarity (*d* = 0.56), and Anxiety Control (*d* = 0.58) (all *p* < 0.05). PCA yielded a four-component structure comprising Willpower (39.13% of explained variance), Psychological Skills (29.49%), Anxiety Level (17.77%), and Tenacity (13.62%). A weighted composite score derived from normalized component loadings significantly differentiated elite from non-elite mountaineers (Fisher–Freeman–Halton exact test, *p* < 0.001).

**Conclusion:**

As an exploratory model-development study, these findings provide preliminary evidence for a willpower-centered, contextually grounded psychological framework and its discriminative utility, offering a preliminary basis for research and training-oriented psychological profiling in elite high-altitude mountaineering.

## 1 Introduction

High-altitude environments (≥2,500 m) are home to approximately 140 million people worldwide and represent extreme ecological contexts in which occupational groups—such as professional mountaineers and high-altitude guides—face persistent and systemic health risks. Prolonged exposure to hypobaric hypoxia has been shown to induce structural damage to the central nervous system (Zhang and Zhang, 2022), contributing to cognitive decline (Zhang et al., 2022), emotional dysregulation (Chen et al., 2020; de Aquino Lemos et al., 2012), and an increased prevalence of chronic mountain sickness (CMS) (Zou et al., 2022). Beyond individual health consequences, these impairments directly affect judgment, coordination, and decision-making under risk, thereby increasing the likelihood of fatal incidents in high-altitude operations. During the 2019 Mount Everest “traffic jam” incident, for example, hypoxia-related decision-making errors were reported to have contributed to 11 fatalities, illustrating the lethal interaction between environmental stressors and human psychological limits (Liu, 2019). These risks extend beyond individual survival to threaten mountain tourism economies, emergency response systems, and labor health in border and plateau regions. Accordingly, precision intervention strategies grounded in localized psychological data are urgently needed.

High-altitude stressors reshape psychological traits through neuroendocrine and neurocognitive pathways. Although the underlying physiological mechanisms of hypoxia are broadly universal, the manifestation, interpretation, and regulation of psychological responses are likely mediated by cultural norms and social values—a dimension that remains insufficiently examined. Hypoxia impairs prefrontal–limbic connectivity, suppressing executive control and emotional regulation (Aboouf et al., 2023), while acute oxygen deprivation reduces attentional resources and elevates risk-decision thresholds (Pun et al., 2018; Meng et al., 2023). These processes help explain the frequently described “double-edged sword effect” of mental toughness (MT), whereby traits that support perseverance and goal pursuit may simultaneously increase risk exposure in extreme environments (Yong et al., 2025; Eikeland et al., 2025). In elite mountaineering—where performance and survival are inseparable—a nuanced understanding of these psychological determinants is therefore essential.

Although successful summit attempts on Mount Everest have been consistently associated with high levels of mental toughness (Burke and Orlick, 2003; Swann et al., 2016; Crust et al., 2016), empirical evidence also indicates that approximately 17% of climbers ignore safety warnings due to excessive perseverance or goal fixation. Existing MT research, however, remains largely rooted in Western, individualistic cultural contexts and struggles to account for the collective resilience patterns observed among Chinese mountaineers, which are often driven by shared goals, moral obligation, and group responsibility (Chen et al., 2023; Maher and Potter, 2001; You et al., 2010). A frequently cited example is the case of Xia Boyu, whose self-sacrificial assistance to teammates—despite severe physical consequences—illustrates how collectivist values can profoundly shape risk-related decision-making. Such culturally embedded behavioral patterns are rarely captured by Western-derived dominant psychological frameworks, which tend to emphasize individual achievement and self-transcendence (Zhang and Tian, 2024).

Cross-cultural research further highlights systematic differences between Western and Chinese mountaineers. Western climbers commonly frame extreme climbing as a pursuit of individual self-transcendence and personal mastery (Ewert, 2001; Maher and Potter, 2001), whereas Chinese climbers often demonstrate a collectivist orientation characterized by teamwork, duty, and shared responsibility (Tan, 2012; Yao et al., 2020; Yuan, 2013). While collectivist cultures may buffer anxiety through social support and group cohesion, they can also suppress individual emotional disclosure, potentially delaying medical intervention and increasing hidden health risks (Boos et al., 2018; Stück et al., 2005). This “collective resilience–individual risk” paradox underscores the conceptual limitations of applying Western MT frameworks without cultural adaptation, as such models may overestimate individual risk tolerance while underestimating the protective—and sometimes costly—effects of culturally specific behaviors.

Despite growing recognition of these issues, systematic investigations into the psychological characteristics underpinning elite performance in high-altitude mountaineering remain scarce. The primary barrier is methodological rather than theoretical: recruiting sufficiently large and well-defined samples from this rare and geographically dispersed population is exceptionally difficult (Jackman et al., 2020). This limitation has constrained the development of empirically grounded, context-sensitive psychological assessment tools for elite athlete selection, training, and risk management.

Current research on mountaineers’ psychological traits faces three interrelated challenges. First, many existing models emphasize single psychological dimensions—such as coping strategies or personality traits—while neglecting the multidimensional structure of psychological adaptation required in extreme environments. This reductionism limits explanatory power and weakens links to real-world performance. Second, the cross-cultural applicability of mainstream MT frameworks, including Clough’s four-dimensional model, remains contested when applied to Chinese athletes, particularly with respect to culturally salient constructs such as honor, collective achievement motivation, and moral responsibility (You et al., 2010; Clough et al., 2002; You, 2012). Uncritical adoption of these models risks biased evaluations in elite athlete selection. Third, existing evaluation criteria rely heavily on Western-developed scales and lack altitude-specific and occupation-relevant indicators, constraining the development of targeted psychological interventions and equitable health management strategies.

In light of these gaps, and acknowledging the practical constraints of sampling elite mountaineers, the present study was explicitly designed as an exploratory, model-development investigation. The study aimed to: (1) identify the core psychological structure of elite Chinese mountaineers using a PCA-based exploratory structure modeling approach to establish a streamlined and culturally informed assessment framework; (2) quantify psychological differences between elite and non-elite mountaineers to construct a performance-sensitive indicator system relevant to occupational profiling and training support; and (3) develop a preliminary set of culturally informed evaluation criteria for elite performance. We hypothesized that (H1) elite mountaineers would score significantly higher than non-elite mountaineers on willpower-related and psychological skill-based indicators, and (H2) that the emergent psychological component structure would reflect an integration of universal psychological adaptations and culturally specific patterns. By addressing these aims, the study responds directly to United Nations Sustainable Development Goal 3 (SDG 3)— “Ensure healthy lives and promote well-being for all”—by providing cross-cultural empirical evidence to inform health management and performance enhancement strategies for high-altitude occupational populations (United Nations, 2019).

## 2 Materials and methods

### 2.1 Study population and sample treatment

#### 2.1.1 Study design and ethical approval

**Figure 1:**
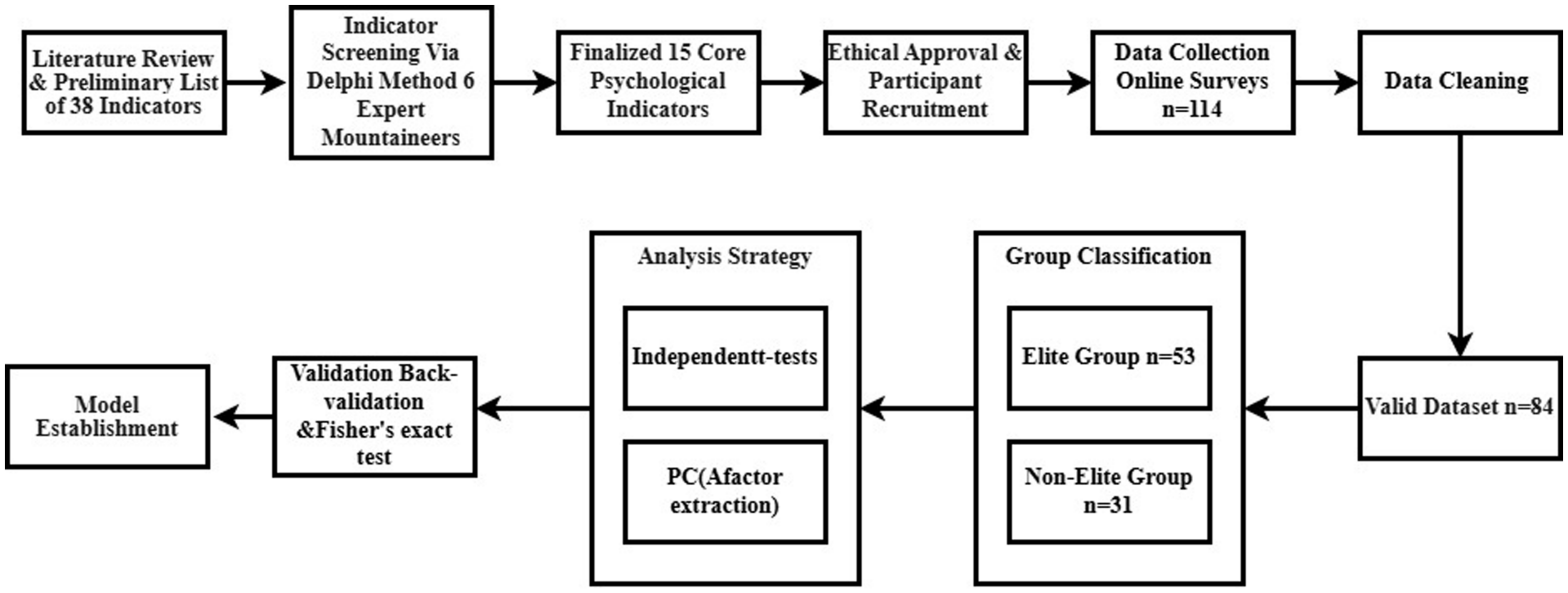
Research workflow diagram: from participant recruitment to result validation.

From September to October 2022, we conducted a cross-sectional, exploratory psychometric study. Its primary aims were to explore the psychological component structure of elite Chinese mountaineers and to establish preliminary evidence for a contextually grounded, culture-informed assessment framework. The overall research procedure—encompassing indicator screening, measurement selection, exploratory structure extraction, and performance-based validation—is summarized in Figure 1. This study was explicitly designed as an initial model-development phase of research.

In the absence of a pre-existing, culturally validated psychological model for this population, an exploratory structure modeling approach was adopted. Although exploratory factor analysis (EFA) is often used as an umbrella term in applied psychology, the present study explicitly employed principal component analysis (PCA) as a data-driven method for exploratory structure extraction, focusing on component identification rather than confirmatory latent variable modeling. Consequently, confirmatory factor analysis (CFA) was reserved for future validation studies with larger, independent samples.

The research protocol received ethical approval from the Academic Integrity and Research Ethics Committee of China University of Geosciences (Wuhan) in June 2022. Upon accessing the electronic questionnaire, all participants were required to review an informed consent form that detailed the study’s purpose, anonymization procedures, data confidentiality safeguards, and the terms of voluntary participation. Access to the questionnaire interface was granted only after participants confirmed their agreement.

To provide preliminary validation evidence within the constraints of this exploratory phase, we incorporated a performance-based known-groups comparison between elite and non-elite mountaineers, a recognized method in early-stage instrument development. Finally, it is important to note that recruiting elite high-altitude mountaineers poses a significant methodological challenge due to their small, geographically dispersed population. The resulting sample size (N = 84) is consistent with those reported in other psychological studies of elite Himalayan and Tibetan Plateau climbers, thereby supporting its use in this preliminary, hypothesis-generating investigation. To reduce potential misinterpretation, all findings are explicitly framed as exploratory and model-developing rather than confirmatory.

#### 2.1.2 Participant recruitment and group classification

A total of 114 questionnaires were administered through a targeted online recruitment campaign, distributed via licensed mountaineering organizations and official athlete networks. Following a rigorous data-cleaning protocol, 84 valid responses were retained for analysis. Participants were classified into elite and non-elite groups based on the Athlete Technical Grade Standard (Department of Athletics, General Administration of Sport [GAS], 2021), the official national benchmark for competitive mountaineering proficiency. The elite group (*n* = 53) consisted of athletes holding at least a national first-grade qualification. Recruitment efforts were concentrated in western China, the primary hub for high-altitude expeditions, with supplementary outreach in northern and central provinces. All elite participants had verified experience summiting peaks above 7,500 m, a recognized threshold for expert high-altitude climbing. The non-elite comparison group (*n* = 31) was composed of mountaineering enthusiasts, predominantly university sports majors, with experience limited to peaks of 5,000–6,000 m, thereby establishing a clear performance contrast for known-groups validation. The recruitment of elite-level participants was inherently challenging due to the small population size and restricted access governed by mountaineering authorities. The final sample size is consistent with those achieved in prior psychological studies of this specialized cohort. All recruitment and grouping procedures were predetermined to ensure methodological transparency. Elite/non-elite classification was determined *a priori* and was not adjusted during analysis.

#### 2.1.3 Data cleaning procedures

All collected questionnaires underwent a multi-stage data quality control procedure to ensure measurement accuracy and psychometric validity. Responses were screened for completeness, internal consistency, and response patterns indicative of inattention or misunderstanding. This process was particularly critical given the linguistic and educational diversity of the national mountaineering cohort. A subset of initial responses—primarily from climbers for whom Mandarin was not a primary language—demonstrated difficulty comprehending abstract psychological terminology and were therefore excluded to prevent systematic measurement bias.

Although 114 questionnaires were initially collected, the strict application of these quality-control criteria resulted in 84 valid cases being retained for final analysis. This decision prioritized data integrity over sample quantity, a recognized and necessary practice in psychological research involving rare or hard-to-access athletic populations. Consequently, the final sample size is methodologically justified and aligns with the sample ranges reported in prior studies of elite high-altitude climbers.

In addition, scale-specific validity checks were applied when available. For the Competitive Cognitive Trait Anxiety Inventory (CCTAI-C), responses failing the embedded lie-detection criteria (i.e., the sum score of lie items < 7 indicating insufficient response credibility) were treated as invalid and excluded according to the instrument guideline. For the BTL-YZ-1.1 scale, questionnaires containing predefined invalid patterns (e.g., specific impossible full-score combinations reported in the original usage rules) were removed.

#### 2.1.4 Demographic characteristics

Elite Group: Participants ranged from 21 to 44 years old (*M* = 32.83 ± 7.07), and most held junior high or high school diplomas. The participants’ occupations included professional mountaineers and high-altitude guides, with an average of 12.96 years (±6.37) of high-altitude climbing experience. Non-Elite Group: Ages ranged from 22 to 52 years old (*M* = 28.90 ± 7.91). Most were undergraduate or graduate students of sports disciplines, with an average climbing tenure of 4.36 years (± 3.81). Women constituted just 9.6% of the final sample.

#### 2.1.5 Note on gender representation

The underrepresentation of female participants (*n* = 8/84) is consistent with global mountaineering demographics (Pomfret and Doran, 2015), and this finding also warrants further consideration. Women may be more likely to use disengagement coping strategies in high-stress scenarios (Andrews, 2014). Future studies should increase the number of female participants to improve generalisability. Given the small number of women, we did not conduct gender-stratified inferential analyses in the present study.

### 2.2 Screening of psychological indicators and selection of measurement tools

#### 2.2.1 Indicator screening

A preliminary list of 38 psychological indicators was compiled through literature review and the Delphi Method. Six elite mountaineers with master’s degrees were recruited to rate the importance of these indicators using a six-point scale (0, 1, 3, 6, 10, 15). Based on predefined screening criteria (a total score ≥ 60), 16 indicators were included in the study. Semantic analysis merged redundant items (e.g., Belief Certainty and Self-Confidence), ultimately identifying 15 core psychological indicators related to occupational health (see Table 1 for details). Following indicator screening and semantic consolidation, all retained indicators were subjected to empirical testing. Only those demonstrating significant performance discrimination between elite and non-elite mountaineers were subsequently entered into component extraction and evaluation model construction, thereby minimizing conceptual redundancy and enhancing criterion relevance.

**Table 1:** Psychological indicator screening results of mountaineers.

Psychological indicator	Score
Mental Resilience	80
Challenge-Skill Balance	65
Attention	80
Self-Confidence	71
Temperament Type	52
Clear Feedback	58
Altered Perception of Time	25
Sport Performance Improvement	30
Sport Mental Fatigue	36
Injury Anxiety	71
Sport performance anxiety	65
Financial Risk Propensity	25
Moral Risk Propensity	29
Decisiveness	90
Self-Discipline	68
Mental Preparation	76
Motivation	53
Anxiety Control	68
Loss of Self-Consciousness	44
Enjoyment	21
Effort Orientation	53
Individual Failure Anxiety	25
Social Evaluation Anxiety	27
Failure Anxiety	36
Physical Risk Propensity	53
Focus on Current Task	76
Goal Clarity	85
Belief Certainty	81
Team Cohesion	65
Autotelic Experience	40
Merging of Action and Awareness	48
Sense of Control	65
Perceptual Experience	36
Social Recognition	26
Social Expectation Anxiety	20
Sports Preparation Anxiety	62
Teammate Competence Anxiety	25
Social Risk Propensity	22

#### 2.2.2 Measurement tools

A multidimensional psychological assessment system was implemented, utilizing exclusively validated Chinese versions of all instruments. It is important to note that no new translation or cross-cultural adaptation was undertaken for this study; the English descriptions provided herein are for academic reporting purposes only. The mapping of original scale dimensions to the core psychological indicators of this study was conducted as follows:

(1) BTL-YZ-1.1 Elite Athlete Volition scale (*BTL-YZ-1*) (Liang et al., 2005): The original six dimensions of this Chinese-developed scale were aligned with the study’s core indicators. Specifically, the *Tenacity* and *Perseverance* dimensions were combined to form a composite measure of mental resilience. The *Self-Contro*l dimension was used to assess self-discipline, while the *Decisiveness*, *Belief Certainty*, and *Goal Clarity* dimensions were used to directly measure their respective indicators. Item scoring followed the original instrument rules, including both forward- and reverse-keyed items for relevant subscales (e.g., Decisiveness), to preserve construct validity.

(2) Psychological Skills Inventory for Sport (*PSIS-C*) (Cox and Liu, 1993): Select dimensions from the validated Chinese version were mapped to four core indicators. The *Attentional Focus* dimension measured attention, and the Team Importance dimension was adapted to assess team cohesion in a non-competitive expedition context. The *Mental Preparation* and *Anxiety Control* dimensions were directly used to measure their corresponding indicators.

(3) Competitive Cognitive Trait Anxiety Inventory (*CCTAI-C*) (Ye et al., 2000): The three dimensions of this validated Chinese inventory—*Sport Preparation Anxiety*, *Sport Performance Anxiety*, and *Injury Anxiety*—were directly mapped to their respective core indicators. In this instrument, higher scores indicate higher anxiety; therefore, these indicators were treated as “low-optimal” dimensions in interpretation and in the construction of evaluation criteria.

(4) Flow State Scale (Chinese version; Jackson and Marsh, 1996): From the validated Chinese version, the *Concentration on Task at Hand* dimension was used to measure focus on immediate tasks, while the *Sense of Control* and *Challenge–Skill Balance* dimensions independently assessed their respective indicators.

All scales demonstrated satisfactory reliability for exploratory research, with an overall Cronbach’s *α* ≥ 0.80 and subdimension α values ranging from 0.60 to 0.80, thereby meeting psychometric standards for preliminary model development in specialized athletic populations. Detailed information on each instrument, including item numbers, response scales, reverse-coded items, and scoring procedures, is provided in Supplementary Table S2.

### 2.3 Statistical analysis

All statistical analyses were performed using SPSS 26.0. Given the cross-sectional design, causal inferences cannot be derived from the data. Prior to inferential analysis, the distributional normality of all psychological indicators was examined using the Shapiro–Wilk test. The results indicated no statistically significant deviation from normality across indicators (all *p* > 0.05). Although several Shapiro–Wilk *p*-values were relatively close to the 0.05 threshold, skewness and kurtosis indices were additionally inspected, and full normality diagnostics are reported in Supplementary Table S1.

Given the moderate sample size and balanced group structure, independent-samples *t*-tests were considered appropriate, as such procedures are generally robust to minor departures from normality under these conditions. Differences in psychological profiles between elite and non-elite mountaineers were examined using independent samples t-tests (*α* = 0.05). Homogeneity of variance was assessed using Levene’s test; when the assumption was violated, Welch’s *t*-test was applied. Effect sizes were quantified using *Cohen’s d* to support practical interpretation.

As this study represents the initial, model-development phase of research in a population lacking an established psychological framework, a PCA-based exploratory structure modeling approach was adopted to identify psychologically meaningful component structures rather than to confirm a predefined latent model.

Exploratory Structure Modeling Using Principal Component Analysis (PCA). The primary objective of this study was to explore the psychological component structure of elite Chinese mountaineers in the absence of a pre-existing, culturally validated theoretical model. Accordingly, an exploratory structure modeling strategy was adopted. While exploratory factor analytic techniques are frequently referenced in psychometric research, the present study employed principal component analysis (PCA) as the extraction method, emphasizing data-driven component identification rather than confirmatory latent variable modeling. Confirmatory factor analysis (CFA) is therefore reserved for future studies designed to test the stability and generalizability of this preliminary structure in independent samples.

The Kaiser–Meyer–Olkin (KMO) measure of sampling adequacy was 0.638, indicating lower-bound adequacy for exploratory PCA; Bartlett’s test of sphericity was significant (*p* < 0.001) (Hair et al., 2010). Given the exploratory, model-development purpose and the specialized nature of the sample, this KMO level supports preliminary component identification, but the stability of the extracted component structure should be confirmed in larger, independent samples.

Principal component analysis (PCA) was employed as the extraction method. To achieve a scientifically interpretable and parsimonious component structure, an orthogonal rotation was performed using the Varimax method. The number of components to retain was determined by selecting those with eigenvalues greater than 0.9. This slightly relaxed criterion was adopted to retain psychologically meaningful variance in a specialized sample, while prioritizing interpretability over strict adherence to the eigenvalue-of-1 rule, thereby achieving a comprehensive cumulative variance explanation of 71.668%.

Post-Hoc Power and Evaluation Criteria. A post-hoc power analysis was conducted using G*Power 3.1. The analysis indicated that the study was adequately powered (1-*β* = 0.96) to detect the large effect sizes observed in our key group comparisons. However, it was underpowered to detect more subtle effects (Faul et al., 2009). Finally, to establish a practical evaluation tool, a weighted composite score was calculated based on normalized factor loadings. Individual indicator grades and overall competence levels were classified using the Deviation Method to establish a transparent, replicable evaluation standard.

#### 2.3.1 Formulas for weight calculation

Normalization was performed based on factor loading coefficients, with the formulas defined as follows:

(1) Comprehensive Weight of Each Indicator:

$$W_{i}=\sum_{j=1}^{k}\frac{|a_{\mathrm{ij}}|}{S_{j}}$$

Where *Wi*: Comprehensive weight of the *i*-th indicator, *aij*: Factor loading of the *i*-th indicator on the *j*-th principal component, and *Sj*: Sum of absolute factor loadings for the *j*-th component.

(2) Weight Percentage:

$$P_{i}=\frac{W_{i}}{\sum W_{i}}$$

Where *Pi*: Percentage weight of the *i*-th indicator, and ∑*Wi*: sum of all comprehensive weights.

## 3 Results

### 3.1 Comparison of psychological indicators between elite and non-elite mountaineers

Independent-samples t-tests revealed significant differences between elite and non-elite mountaineers on multiple psychological indicators (see Table 2). Elite climbers scored significantly higher on Perseverance (*t* = 2.90, *p* < 0.01), Tenacity (*t* = 2.14, *p* < 0.05), Decisiveness (*t* = 2.34, *p* < 0.05), Goal Clarity (*t* = 2.47, *p* < 0.05), Anxiety Control (*t* = 2.57, *p* < 0.05), Attentional Focus (*t* = 2.41, *p* < 0.05), and Sense of Control (*t* = 2.03, *p* < 0.05). No significant group differences were observed in Self-Control, Belief Certainty, Team Importance, Challenge–Skill Balance, or Focus on the Current Task. For the low-optimal indicator domain (Sport Cognitive Trait Anxiety), significant differences were found in Sport Preparation Anxiety (*t* = −2.01, *p* < 0.05) and Injury Anxiety (*t* = −2.85, *p* < 0.01), with higher anxiety levels consistently reported among non-elite mountaineers. No significant difference was detected for Sport Performance Anxiety.

**Table 2:** Psychological indicators differentiating elite and non-elite mountaineers.

Category	Indicator	Elite group (Mean ± SD)	Non-elite group (Mean ± SD)	Cohen’s *d*	*t*
Willpower traits	Perseverance	40.81 ± 4.46	37.81 ± 4.81	0.66	2.90**
	Tenacity	36.47 ± 4.56	34.42 ± 3.66	0.48	2.14*
	Decisiveness	28.08 ± 3.77	26.03 ± 4.07	0.53	2.34*
	Self-Control	28.13 ± 4.39	27.29 ± 3.21	0.21	0.93
	Belief Certainty	25.06 ± 3.76	24.68 ± 3.49	0.10	0.46
	Goal Clarity	25.87 ± 3.40	24.10 ± 2.72	0.56	2.47*
Sports psychological skills	Anxiety Control	33.75 ± 4.55	31.35 ± 3.29	0.58	2.57*
	Attentional Focus	19.11 ± 3.57	17.45 ± 2.71	0.51	2.41*
	Mental Preparation	24.26 ± 2.52	22.71 ± 2.71	0.60	2.65**
	Team Importance	27.06 ± 2.88	26.32 ± 2.57	0.27	1.17
Sports cognitive trait anxiety	Sport Preparation Anxiety	15.86 ± 3.54	17.42 ± 3.18	0.45	−2.01*
	Sport Performance Anxiety	14.66 ± 3.03	13.74 ± 2.91	0.31	1.36
	Injury Anxiety	9.96 ± 2.39	11.22 ± 1.67	0.59	−2.85**
Flow experience	Challenge-Skill Balance	14.60 ± 3.06	13.81 ± 2.73	0.27	1.20
	Concentration on Task at Hand	15.47 ± 2.00	14.84 ± 1.99	0.32	1.41
	Sense of Control	14.47 ± 2.67	13.26 ± 2.58	0.46	2.03^*^

Cohen’s *d* represents effect size: 0.2 = small, 0.5 = medium, 0.8 = large. *p < 0.05, **p < 0.01.

Full normality diagnostics (Shapiro–Wilk *W* and *p*-values, skewness, and kurtosis) are provided in Supplementary Table S1.

*Cohen’s d* values indicated moderate to large effect sizes (*d* = 0.46–0.66) for indicators showing significant group differences, whereas non-significant indicators exhibited small effect sizes (*d* = 0.10–0.32). Taken together, these results provide robust preliminary evidence for the criterion-related validity of the selected indicators in differentiating performance levels, highlighting distinct psychological profiles among elite mountaineers, particularly in Willpower-related attributes (e.g., Perseverance, Tenacity) and Psychological Skills (e.g., Anxiety Control, Attentional Focus). On the basis of these group-level distinctions, further multivariate analysis was conducted to elucidate the latent psychological structure underlying elite mountaineering performance. Accordingly, principal component analysis (PCA) was performed to identify the core components characterizing psychological resilience in extreme environments such as the “Death Zone” (above 8,000 m).

### 3.2 Principal component analysis results of psychological characteristics of elite mountaineers

Sensitive indicators, defined as representative psychological metrics that are closely associated with sport-specific performance and selection outcomes (Zeng, 1992), serve as critical references for athlete evaluation. Among the 15 psychological indicators analyzed, 10 indicators—Perseverance, Tenacity, Goal Clarity, Anxiety Control, Attentional Focus, Mental Preparation, Sense of Control, Decisiveness, Sport Preparation Anxiety, and Injury Anxiety—demonstrated significant group differences and were therefore retained for component extraction (see Table 2). Principal component analysis (PCA) was conducted to examine the structural characteristics of these indicators. The Kaiser–Meyer–Olkin (KMO) measure of sampling adequacy was 0.638, and Bartlett’s test of sphericity was significant (χ^2^, *p* < 0.001), indicating that the correlation matrix was suitable for component analysis.

To develop a comprehensive and psychologically interpretable model, four components with eigenvalues greater than 0.9 were retained. Although this criterion is less conservative than the traditional eigenvalue-of-1 rule, it was adopted in this exploratory, model-development context to retain potentially meaningful variance in a specialized sample. Together, these four components accounted for a cumulative variance explanation of 71.668% (see Table 3).

**Table 3:** Eigenvalues, variance explained, cumulative variance explained, and contribution rates of psychological indicators (elite group).

Component	Eigenvalue	Variance explanation (%)	Cumulative variance explained (%)	Contribution after rotation (%)
1	2.804	28.042	28.042	39.128
2	2.113	21.131	49.173	29.485
3	1.273	12.733	61.907	17.767
4	0.976	9.761	71.668	13.620

An orthogonal rotation using the Varimax method was applied to simplify the component structure and enhance interpretability. As shown in Table 4, the first component comprised Perseverance, Goal Clarity, Mental Preparation, and Sense of Control, and was labeled the Willpower Component. The second component consisted of Anxiety Control and Attentional Focus and was labeled the Psychological Skills Component. The third component included Sport Preparation Anxiety, Injury Anxiety, and Decisiveness and was labeled the Anxiety Level Component. The fourth component was exclusively characterized by Tenacity and was labeled the Tenacity Component. To determine the relative contribution of each component to the overall psychological structure of elite mountaineers, contribution rates were calculated by dividing the variance explained by each component by the total cumulative variance after rotation. The Willpower Component accounted for the largest proportion of explained variance (39.128%), followed by the Psychological Skills Component (29.485%), the Anxiety Level Component (17.767%), and the Tenacity Component (13.620%). These results indicate a clearly differentiated, four-component psychological architecture underlying elite high-altitude mountaineering performance (see Table 3).

**Table 4:** Orthogonal rotation component loadings matrix of psychological indicators (elite group).

Indicators	Willpower component	Psychological skills component	Anxiety level component	Tenacity component
Perseverance	**0.747**	–	–	–
Goal clarity	**0.708**	–	–	–
Mental preparation	**0.689**	–	–	–
Sense of control	**0.791**	–	–	–
Anxiety control	–	**0.903**	–	–
Attentional focus	–	**0.906**	–	–
Decisiveness	–	–	**−0.603**	–
Sport preparation anxiety	–	–	**0.743**	–
Injury anxiety	–	–	**0.762**	–
Tenacity	–	–	–	**0.913**
Eigenvalue	2.804	2.113	1.273	0.976

Only factor loadings ≥ 0.60 are reported; “–” indicates loadings < 0.60. Bold values indicate the primary factor loadings retained for interpretation of each component.

### 3.3 Evaluation criteria for psychological indicators of elite mountaineers

Based on the extracted component structure, evaluation criteria were constructed following established principles for resilience and performance assessment (Luthar et al., 2000). The 10 sensitive indicators (see Table 2) were classified into high-priority and low-priority categories according to their component loadings and functional relevance. Each indicator was subsequently categorized into five performance levels—Excellent, Good, Moderate, Poor, and Very Poor—with corresponding quantitative scores assigned to each level. The Deviation Method (±1.28 *s* and ±0.52 *s*) was applied to establish classification thresholds for individual indicators, thereby ensuring statistical transparency and replicability. Indicator weight percentages were determined based on normalized component loadings (Wang, 1992), reflecting their relative importance within the composite psychological structure.

Individual psychological scores were calculated according to the indicator-specific criteria, and comprehensive evaluation scores were obtained by multiplying each indicator score by its corresponding weight and summing across indicators. The detailed scoring criteria and threshold values are presented in Tables 5–8.

**Table 5:** Single and comprehensive scoring criteria.

Percentile method (in statistics)	(Math.) discrete method		Score (level)
	High-priority indicators	Low-priority indicators	
>P_90_	≥ *x̄* + 1.28 *s* (High-Priority)	≤ *x̄* − 1.28 *s*	100 (Excellent)
P_70_-P_90_	*x̄* + 0.52 *s* to *x̄* + 1.28 *s*	*x̄* − 1.28 *s* to *x̄* − 0.52 *s*	80 (Good)
P_30_-P_70_	*x̄* ± 0.52 *s*	*x̄* ± 0.52 *s*	60 (Moderate)
P_10_-P_30_	*x̄* − 1.28 *s* to x̄ − 0.52 *s*	*x̄* + 0.52 *s* to *x̄* + 1.28 *s*	40 (Poor)
<P_10_	≤ *x̄* − 1.28 *s*	≥ *x̄* + 1.28 *s* (Low-Priority)	20 (Very Poor)

**Table 6:** High-priority indicator evaluation standards (elite group).

Indicators	Excellent	Good	Moderate	Poor	Very poor
Sense of control	≥17.86	15.85–17.85	13.10–15.84	11.08–13.09	≤11.07
Perseverance	≥46.47	43.11–46.46	38.51–42.10	35.16–38.50	≤35.15
Goal clarity	≥30.17	27.62–30.16	24.12–27.61	21.56–24.11	≤21.55
Mental preparation	≥27.64	25.56–27.63	22.29–25.55	21.07–22.28	≤21.06
Attentional focus	≥23.63	20.95–23.62	17.28–20.94	14.59–17.27	≤14.58
Anxiety control	≥39.53	36.10–39.52	31.14–36.09	27.98–31.13	≤27.97
Decisiveness	≥32.84	30.01–32.83	26.14–30.00	23.31–26.13	≤23.30
Tenacity	≥42.25	38.83–42.24	34.12–38.82	30.69–34.11	≤30.68

**Table 7:** Low-priority indicator evaluation standards (elite group).

Indicators	Excellent	Good	Moderate	Poor	Very poor
Sport preparation anxiety	≤11.38	11.39–14.04	14.05–17.69	17.70–20.36	≥20.37
Injury anxiety	≤6.94	6.95–8.73	8.74–11.19	11.20–12.99	≥13.00

**Table 8:** Comprehensive psychological competence evaluation criteria (elite group).

Score type	Excellent	Good	Moderate	Poor	Very poor
Composite score	≥69.90	63.65–69.89	55.11–63.64	48.86–55.10	≤48.85

### 3.4 Back-validation of the comprehensive evaluation criteria for elite mountaineers

To assess the internal validity and discriminatory accuracy of the proposed evaluation system, a back-validation procedure was conducted using the full sample. The validation process consisted of three steps: (1) calculating individual indicator scores based on the established evaluation criteria; (2) computing comprehensive psychological scores using weighted aggregation; and (3) classifying overall psychological competence into five predefined levels (Excellent, Good, Moderate, Poor, and Very Poor).

The composite evaluation score derived from the four-component model demonstrated strong known-groups validity. As shown in Table 9, the distribution of psychological competence levels differed significantly between elite and non-elite groups (Fisher–Freeman–Halton exact test, *p* < 0.001). Notably, no non-elite mountaineers were classified in the “Excellent” category, whereas elite mountaineers were predominantly distributed across higher competence levels. These findings provide preliminary within-sample support for the evaluation framework’s known-groups differentiation; independent external validation is required before practical application. A comprehensive summary of the validation results is presented in Tables 9, 10.

**Table 9:** Frequency distribution of psychological competence levels (elite vs. non-elite groups).

Group	Excellent	Good	Moderate	Poor	Very poor	Total
Elite group	7	10	21	11	4	53
Non-elite group	0	2	1	13	15	31

**Table 10:** Significance test for differences in level frequency distribution (elite vs. non-elite groups).

Test method	Value	Exact significance (two-sided)
Fisher–Freeman–Halton Exact Test	34.519	*P* < 0.001

## 4 Discussion

### 4.1 Core status of the willpower component: a central element in elite performance

The Willpower Component demonstrated the highest contribution to the total variance (39.13%), indicating its potentially central role within the PCA-based psychological structure identified in this study and highlighting the importance of willpower-related attributes in elite high-altitude mountaineering. This finding is consistent with extensive literature indicating that mental toughness is a central psychological characteristic in mountaineering contexts (Burke and Orlick, 2003; Crust et al., 2018; Jackman et al., 2020). For instance, willpower has been shown to be critical for overcoming physiological distress at high altitudes and for sustaining goal-directed behavior under extreme environmental stress (Crust et al., 2018; Swann et al., 2016). This study further refines the operational composition of willpower by highlighting the prominent performance of Goal Clarity (*M* = 25.87 ± 3.40) and Sense of Control (*M* = 14.47 ± 2.67), which may reflect elite climbers’ ability to maintain cognitive clarity and behavioral regulation in extreme environments (Burns et al., 2020; Crust et al., 2016). These elements should be understood as functionally executable psychological capacities rather than abstract personality traits, enabling elite performers to formulate clear tactical plans under duress and maintain operational autonomy when external support is absent (Ekinci et al., 2025; Davranche et al., 2016)—capacities that are particularly relevant in the “Death Zone.” In high-altitude contexts, these willpower-related capacities may also underpin courageous action under life-threatening conditions, as frequently described in mountaineering literature (Rate et al., 2007; Norton and Weiss, 2009). Such capacities likely facilitate sustained decision-making and risk regulation under extreme hypoxic and environmental stress, where psychological breakdown can have immediate survival consequences. The alignment of the Willpower Component with the “sense of control” and “commitment” dimensions proposed by Clough et al. (2002) provides contextualized empirical support for established mental toughness theory. However, it should be emphasized that the present component structure reflects a PCA-based exploratory configuration rather than a direct validation of an existing latent trait model.

While the overarching component labels (e.g., Willpower, Psychological Skills) may appear analogous to Western-derived frameworks, their constituent elements and empirical configuration in this sample warrant a culturally contextualized interpretation. In other words, similarities may exist at the level of component labels, but not necessarily at the level of meaning and application. For example, the high loading of Goal Clarity within the Willpower Component may not solely reflect individual ambition. In the Chinese mountaineering context, it may also be partially shaped by a collectivist sense of purpose and responsibility toward the team, a motivational orientation frequently documented in qualitative studies of Chinese climbers (Chen et al., 2023; Yao et al., 2020; You et al., 2010). Importantly, cultural variables were not directly measured in this study; therefore, these interpretations should be regarded as theoretically informed explanations rather than empirically tested cultural mechanisms. Similarly, Anxiety Control and Mental Preparation may function not only to optimize individual performance but also to maintain group stability and coordinated action under stress, aligning with collectivist coping patterns described in prior research (Miçooğullari and Kirazci, 2016; Yang et al., 2020). Taken together, these findings suggest that the present model is best characterized as culture-contextualized in interpretation and application, rather than as a direct measurement of cultural constructs.

### 4.2 Synergistic function of the psychological skills component: differentiating elite performance

Within the Psychological Skills Component (29.49%), elite climbers demonstrated significantly higher levels of Anxiety Control (*M* = 33.75 ± 4.55 vs. 31.35 ± 3.29, *p* < 0.05) and Attentional Focus (*M* = 19.11 ± 3.57 vs. 17.45 ± 2.71, *p* < 0.05) compared with non-elite climbers. These results are consistent with Wagstaff and Weston’s (2014) conclusion that attentional focus strategies play a critical role in regulating anxiety under performance pressure.

This synergistic configuration is particularly relevant in elite mountaineering, where optimal arousal regulation and the ability to filter environmental and internal distractions are prerequisites for precise technical execution and complex decision-making. Of note, the between-group effect size for Mental Preparation reached *Cohen’s d* = 0.60 (*M* = 24.26 ± 2.52), underscoring the practical relevance of systematic mental rehearsal in extreme environments. This finding aligns with Burke and Orlick’s (2003) proposition that mental rehearsal enhances adaptability in high-risk and unpredictable contexts.

Rather than implying cultural specificity at the structural level, these results indicate that psychological skills commonly identified in sport psychology may acquire context-dependent functional salience in high-altitude expeditions. Accordingly, the Psychological Skills Component represents a set of modifiable and trainable targets that may be particularly relevant for structured psychological skills training programs aimed at aspiring elite mountaineers (Turchetto et al., 2025).

### 4.3 Characteristics of the anxiety level component: implications for risk management and decision-making

The Anxiety Level Component (17.77%) revealed that elite climbers exhibited significantly lower levels of Sport Preparation Anxiety (*M* = 15.86 ± 3.54 vs. 17.42 ± 3.18, *p* < 0.05) and Injury Anxiety (*M* = 9.96 ± 2.39 vs. 11.22 ± 1.67, *p* < 0.05) compared with non-elite climbers, consistent with prior findings among Mount Everest survivors (Swann et al., 2016). In parallel, the elite group demonstrated a moderate effect size for Decisiveness (*Cohen’s d* = 0.53, *M* = 28.08 ± 3.77).

This configuration suggests a performance-relevant psychological profile characterized by lower anxiety levels co-occurring with higher decisiveness, rather than implying a direct causal relationship between these attributes. These between-group differences are interpreted as performance-linked psychological profiles and should not be construed as causal effects of elite status.

Effective anxiety regulation and decisiveness are widely recognized as essential for risk management in high-altitude environments (Yong et al., 2025), where delayed or irrational decisions can have fatal consequences. The present findings provide preliminary, profile-level evidence that these attributes jointly differentiate elite from non-elite climbers, supporting the relevance of the Anxiety Level Component within the overall evaluation framework. Nevertheless, the predictive validity of this component for real-world safety outcomes requires further longitudinal and external validation.

### 4.4 Distinct role of the tenacity component: sustaining performance under prolonged adversity

Despite accounting for the smallest proportion of explained variance (13.62%), the Tenacity Component demonstrated a meaningful effect size (*Cohen’s d* = 0.48), indicating that elite climbers exhibit particularly strong advantages in contexts involving prolonged physical and psychological adversity. This finding is consistent with Jackman et al.’s (2020) assertion that enduring hardship is a defining feature of mountaineering experience.

The emergence of Tenacity as an independent component, distinct from the broader Willpower Component, is a noteworthy result. While tenacity is often conceptualized as a subdimension of willpower in general psychology, its separation in this study may reflect the unique demands of high-altitude mountaineering, which involves sustained exposure to fatigue, hypoxia, and existential risk over extended periods. This observation resonates with Duckworth et al.’s (2007) conceptualization of grit as perseverance toward long-term goals, distinct from momentary self-control or decision-making capacity. Although single-indicator components should be interpreted cautiously, the high loading (0.913) and conceptual distinctiveness of Tenacity justified its provisional retention in this exploratory phase.

This differentiation may reflect distinct underlying psychological or neurocognitive mechanisms, with acute executive control processes supporting short-term willpower and sustained effort regulation contributing to tenacity during prolonged expeditions, as suggested by previous research (MacDonald et al., 2000; Botvinick et al., 2001; Kerns et al., 2004). However, such interpretations remain speculative and require direct neurocognitive and longitudinal evidence.

Consistent with mountaineering literature emphasizing prolonged suffering as a core experiential element (Jackman et al., 2020; Bassi and Fave, 2010), the present findings suggest that tenacity may represent a particularly salient psychological attribute in extreme expeditionary contexts. Rather than being framed as a non-compensable trait, tenacity should be understood as a critical—but context-dependent—capacity that contributes to sustained engagement and expedition completion. Accordingly, assessing and cultivating tenacity may be valuable for informing readiness profiling and developmental assessment, as well as for the design of long-duration resilience training programs.

## 5 Study limitations

The interpretations of this study’s findings should be contextualized within its overarching aim as an exploratory, model-development investigation and the inherent constraints of its target population. These considerations, while bounding the current work, also chart a clear pathway for future research.

First, regarding the sample size (N = 84), it is imperative to distinguish between statistical ideals and methodological reality in niche populations. While the sample is substantial for this rare cohort and was subjected to rigorous quality control, we acknowledge that it limits the detection of subtle effects, as quantified by our post-hoc power analysis. However, the study was robustly powered (1-*β* > 0.96) to identify the strong, central effects that form the core of the proposed model. Thus, while the sample size limits statistical generalizability, it represents a realistic and methodologically defensible foundation for this initial exploratory phase in a rare and specialized population.

Second, systematic differences between the elite and non-elite groups—including age, educational background, professional status, and years of high-altitude experience—were inherent to the study design and were not statistically controlled. Although these differences reflect real-world performance stratification, they may also contribute to observed psychological profile differences through professional socialization or accumulated experience rather than elite performance status alone. Accordingly, group comparisons should be interpreted as performance-linked psychological profiles rather than causal effects of elite performance per se.

Third, the cross-sectional, exploratory design means that causal inference and external validation via confirmatory factor analysis (CFA) are beyond the scope of this study. We explicitly position this work as the foundational stage in the established psychometric development cycle. The logical and essential next step is to test the stability, factorial structure, and predictive validity of this preliminary component-based model through longitudinal designs and CFA in future, independent cohorts.

Fourth, the gender imbalance (9.6% female) reflects the current demographic landscape of professional high-altitude mountaineering in China. While this limits gender-specific generalizations, it accurately represents the population from which elite performers are currently drawn. Future studies should proactively seek greater gender balance as participation patterns evolve. Given the small proportion of female participants, the stability of the component structure across gender remains unknown and should be examined via measurement invariance testing in future samples.

Fifth, although the present study emphasizes culturally contextualized interpretation, cultural variables (e.g., collectivism, duty orientation, or social obligation) were not directly measured. Accordingly, the designation “culture-informed” refers to the contextual grounding of the model, the use of validated Chinese instruments, and its application within a specific occupational and sociocultural setting, rather than to the empirical testing of cultural mechanisms. Direct examination of cultural pathways remains an important objective for future research.

In addition, several methodological aspects of the exploratory PCA should be noted. The KMO value (0.638) lies at the lower threshold of adequacy, so the stability of the extracted component structure should be verified in larger samples. Because a slightly relaxed retention criterion was used (eigenvalue > 0.9), the possibility of overextraction cannot be entirely excluded; future validation studies may apply alternative retention rules such as parallel analysis. Moreover, the Tenacity component was represented by a single indicator (loading = 0.913) and should therefore be regarded as provisional at this stage, pending replication and potential expansion with additional indicators. Finally, because indicators were screened based on group differences and then used to classify the same dataset, the within-sample validation is subject to potential circularity and requires independent external validation.

Beyond these methodological considerations, although the present study relied on self-report data, future research could enrich the evidence base by integrating physiological biomarkers and objective performance or safety indicators to develop a more multimodal understanding of psychological adaptation in extreme environments. Taken together, these limitations do not detract from the present findings but rather delineate the appropriate evidentiary boundaries of this study and clarify its role as an initial, hypothesis-generating contribution.

## 6 Conclusion

This study, positioned as an essential exploratory investigation, proposes and provides exploratory evidence for a four-dimensional, PCA-based psychological component structure for elite Chinese mountaineers, identifying willpower-related attributes as the central performance-relevant component, supplemented by psychological skills, anxiety regulation, and tenacity. This structure represents an empirically derived profiling framework developed within an exploratory phase, rather than a finalized or fully validated model, and differentiates elite from non-elite performers within a specialized high-altitude context.

The resultant model, coupled with its contextually grounded preliminary evaluation framework, offers a heuristic approach for the assessment and targeted psychological training of elite high-altitude mountaineers—a population previously lacking a dedicated, empirically informed profiling approach. Importantly, the proposed framework is intended as a prototype, supported by internal and known-groups validation only, and should not yet be used as a standalone basis for selection, certification, or high-stakes decision-making.

We explicitly frame this model as an initial step within a broader psychometric development process, necessitated by the substantial challenges inherent in sampling elite high-altitude populations. Its value lies not in its finality, but in providing a testable structural hypothesis and a coherent starting point for subsequent validation. Accordingly, critical next steps include (a) replication in larger and independent samples, (b) confirmatory factor analysis or related structural modeling approaches, and (c) longitudinal and multimodal studies integrating objective performance and safety indicators to establish predictive and external validity.

Within these clearly defined boundaries, the present study contributes a contextually grounded and empirically structured psychological profiling framework, developed using validated Chinese instruments and anchored in the occupational realities of elite high-altitude mountaineering. While cultural mechanisms were not directly tested, the framework offers a meaningful culture-informed foundation for future theoretical refinement, psychometric validation, and applied exploration of psychological functioning in extreme environments.

## Data Availability

The raw data supporting the conclusions of this article will be made available by the authors, without undue reservation.
