# Supplementary material for: A culture-informed psychological model for elite Chinese mountaineers: an exploratory study and development of a performance-based assessment tool
Source: Front Psychol. 2026 Mar 25;17:1746906. doi: 10.3389/fpsyg.2026.1746906 (PMC13056870; doi:10.3389/fpsyg.2026.1746906)
Supplement: Supplementary file 1 [file Supplementary_file_1.docx]

# Supplementary Table

**Table S1.** Normality Diagnostics for Psychological Indicators (Shapiro–Wilk Test).

| Category | Indicator | Skewness | Kurtosis | Shapiro–Wilk W | p-value |
| --- | --- | --- | --- | --- | --- |
| Willpower Traits | Perseverance | −0.216 | 1.284 | 0.968 | 0.076 |
|  | Tenacity | 0.362 | −0.157 | 0.974 | 0.086 |
|  | Decisiveness | −0.025 | 0.425 | 0.985 | 0.455 |
|  | Self-Control | −0.301 | 0.505 | 0.976 | 0.120 |
|  | Belief Certainty | 0.267 | −0.821 | 0.960 | 0.051 |
|  | Goal Clarity | 0.143 | −0.033 | 0.983 | 0.313 |
| Sports Psychological Skills | Anxiety Control | 0.475 | 0.053 | 0.972 | 0.066 |
|  | Attentional Focus | −0.071 | −0.191 | 0.981 | 0.234 |
|  | Mental Preparation | 0.156 | −0.474 | 0.972 | 0.066 |
|  | Team Importance | 0.227 | −0.987 | 0.961 | 0.053 |
| Sports Cognitive Trait Anxiety | Sport Preparation Anxiety | 0.103 | −0.394 | 0.980 | 0.232 |
|  | Sport Performance Anxiety | −0.014 | −0.628 | 0.975 | 0.108 |
|  | Injury Anxiety | −0.357 | 0.421 | 0.962 | 0.058 |
| Flow Experience | Challenge-Skill Balance | −0.303 | −0.429 | 0.970 | 0.053 |
|  | Concentration on Task at Hand | −0.023 | −0.394 | 0.970 | 0.056 |
|  | Sense of Control | 0.054 | −0.577 | 0.977 | 0.128 |

Normality was assessed using the Shapiro–Wilk test. All psychological indicators showed no significant deviation from normality (*p* > 0.05). Reported p-values are asymptotic two-tailed estimates as provided by SPSS (version 26.0).

**Table S2.** Measurement Instruments and Scoring Rules.

| Category | Psychological Indicator (Used in This Study) | No. of Items | Response Scale | Score Range | Reverse-Coded Items | Interpretation |
| --- | --- | --- | --- | --- | --- | --- |
| BTL-YZ-1.1 Elite Athlete Volition Scale | Perseverance | 8 | 1–7 Likert | 8–56 | Yes | Higher scores indicate stronger perseverance |
|  | Tenacity | 8 | 1–7 Likert | 8–56 | Yes | Higher scores indicate stronger tenacity |
|  | Decisiveness | 6 | 1–7 Likert | 6–42 | Yes | Higher scores indicate greater decisiveness |
|  | Self-Control | 6 | 1–7 Likert | 6–42 | Yes | Higher scores indicate stronger self-control |
|  | Belief Certainty | 5 | 1–7 Likert | 5–35 | Yes | Higher scores indicate stronger belief certainty |
|  | Goal Clarity | 5 | 1–7 Likert | 5–35 | Yes | Higher scores indicate clearer goal orientation |
| Psychological Skills Inventory for Sport (PSIS-C) | Anxiety Control | 10 | 1–5 Likert | 10–50 | No | Higher scores indicate better anxiety regulation |
|  | Attentional Focus | 6 | 1–5 Likert | 6-30 | No | Higher scores indicate better attentional focus |
|  | Mental Preparation | 6 | 1–5 Likert | 6-30 | No | Higher scores indicate stronger mental preparation |
|  | Team Importance | 7 | 1–5 Likert | 7-45 | No | Higher scores indicate stronger team orientation |
| Competitive Cognitive Trait Anxiety Inventory (CCTAI-C) | Sport Preparation Anxiety | 6 | 1–4 Likert | 6–24 | No | Higher scores indicate higher anxiety |
|  | Sport Performance Anxiety | 5 | 1–4 Likert | 5–20 | No | Higher scores indicate higher anxiety |
|  | Injury Anxiety | 4 | 1–4 Likert | 4–16 | No | Higher scores indicate higher anxiety |
| Flow State Scale (Chinese version) | Challenge-Skill Balance | 4 | 1–5 Likert | 4–20 | No | Higher scores indicate stronger flow experience |
|  | Concentration on Task at Hand | 4 | 1–5 Likert | 4–20 | No | Higher scores indicate stronger task focus |
|  | Sense of Control | 4 | 1–5 Likert | 4–20 | No | Higher scores indicate stronger perceived control |

All instruments were validated Chinese-language versions. Scoring procedures, including reverse-coded items and invalid-response criteria (e.g., lie-detection items in the CCTAI-C), strictly followed original instrument guidelines. Higher scores consistently reflect stronger psychological attributes, except for anxiety-related indicators, where higher scores indicate greater anxiety.
